# TikTok Tracheostomy Video Analysis of Quality, Credibility, and Readability

**DOI:** 10.7759/cureus.60548

**Published:** 2024-05-18

**Authors:** Gianfranco Galantini, Ruwaa Samarrai, Amy Hughes, Katherine Kavanagh

**Affiliations:** 1 Department of Otolaryngology-Head and Neck Surgery, University of Connecticut, Farmington, USA; 2 Department of Otolaryngology-Head and Neck Surgery, University of Connecticut Health, Farmington, USA; 3 Department of Otolaryngology, Connecticut Children's Medical Center, Hartford, USA

**Keywords:** credibility, quality, tiktok, social media, tracheostomy

## Abstract

Objective

The goal of this study is to analyze the quality, credibility, and readability of videos on TikTok related to tracheostomy in order to assess the adequacy of the information for patient and parental education purposes.

Study design

This was a cross-sectional analysis of online content.

Methods

The social media platform TikTok was explored for videos related to tracheostomy. The search function was utilized with multiple hashtags related to tracheostomy and videos were reviewed and scored for quality, credibility, and readability. Each of the videos was assessed using the DISCERN criteria, JAMA benchmark, and readability score based on text either presented in the video or written in the caption. Pearson’s correlation coefficient was calculated for each of the studied parameters.

Results

The TikTok search bar was queried using multiple hashtags, including “#trach,” “#tracheostomy,” “#trachea,” and “#tracheotomy” for relevant videos from October 14 to October 15, 2021. Overall, 60 videos were selected for complete review and analysis. The total views for all related videos analyzed was 17,712,281. The total likes were 693,812. The videos were primarily posted by non-healthcare professionals making up approximately 72% of all videos. Videos created by physicians generated 63% of all views. The average DISCERN score for each video was 24.83 out of 75. The average Flesch Reading Ease score was 70.59 and the average Flesch-Kincaid Grade level was 5.5. There was a positive DISCERN score and views with R = 0.255 (p = 0.049), positive correlation between DISCERN and likes R = 0.334 (p = 0.009), positive correlation between DISCERN and JAMA R = 0.56 (p=<0.0001), positive correlation between DISCERN and Flesch-Kincaid Grade Level R = 0.330 (p=0.010) and a negative correlation between DISCERN and Flesch Reading Ease Score R = -0.337 (p=0.009). There was also a statistically significant positive correlation between JAMA and Flesch-Kincaid Grade Level R = 0.260 (p=0.045).

Conclusion

Overall, the quality of the videos on TikTok regarding tracheostomy rated poorly on the DISCERN quality index but included text that was fairly easy to read. Currently, medical videos on TikTok do not meet the quality metrics needed to properly educate the public and should not be used as a primary resource.

## Introduction

Social media has a staggering effect on our society today with up to 72% of the public participating in at least one social media platform [1]. The most prominent platforms include YouTube, Netflix, and TikTok [2]. Of these applications, TikTok is one of the newest and most rapidly growing websites. TikTok allows users to incorporate music, text, and other special effects into short, less than 60-second videos. TikTok is unique in that users upload a variety of short videos ranging from educational to entertainment. The TikTok algorithm customizes the “For You” page (homepage), as it learns what the user likes with the intent of keeping the user engaged for longer [3]. TikTok is available in over 150 countries, has over 1 billion users, and has been downloaded over 200 million times in the U.S. alone. The percentage of U.S.-based TikTok users by age is as follows: 10-19-year-olds comprise 32.5% (325 million), 20-29 comprise 29.5% (295 million), 30-39 comprise 16.4% (164 million), 40-49 comprise 13.9% (139 million), and 50+- 7.1% (7.1 million) [4]. Multiple studies have shown that TikTok is a potent channel to inform users of health-relevant information [1,3,5,6]. However, the health information provided in the TikTok videos does not always meet the necessary standards [7].

Studies evaluating online videos for the purpose of medical education in otolaryngology have been published, however, this is one of the first studies aimed at assessing the quality of information regarding a specific otolaryngology-related topic on TikTok [8-10]. This study assessed the TikTok content on tracheostomy, with a focus on tracheostomy indications and maintenance. In the pediatric population, patients with tracheostomies often rely on their tracheostomy tube for long-term survival due to the need for long-term mechanical ventilation or to bypass a level of airway obstruction above the tracheostomy tube [11].

Due to the complexity of this patient population, children, parents, and caregivers may turn to the Internet to learn more about tracheostomy, especially with regard to purpose and long-term maintenance [12]. For this reason, providers need to understand the popular content readily available to the public, as patients may need to be educated and redirected to higher value and more accurate sources of information [13]. By understanding the content that a patient or caregiver may see, providers will be better prepared to answer questions and clarify potential misinformation.

## Materials and methods

Exemption from human subjects research was determined by the Institutional Review Board of Connecticut Children’s. Multiple hashtags were selected as search phrases within the application, TikTok. The hashtags utilized were “#trach,” “#tracheostomy,” “#trachea”, and “#tracheotomy”. Exclusion criteria consisted of videos not related to one of the aforementioned search terms, duplicate videos, videos written or spoken in a non-English language, and any video with no text or caption. A total of 60 videos were selected for review. Each post was analyzed and scored for quality, credibility, readability, poster credentials, and post popularity as primary outcome measures. Posts were randomly selected for a second reviewer and inter-rater reliability was determined using intra-class correlation and percent agreement.

The quality of the videos was analyzed using the DISCERN scoring system. DISCERN is a validated scoring tool that consists of 15 questions plus an overall quality rating [14]. Each of the total 16 questions is rated on a scale of 1-5 with a maximum score of 75. In terms of the categorization of the DISCERN score, excellent is denoted as 63 to 75 points, good as 51 to 62 points, fair as 39 to 50 points, poor is 27 to 38 points, and very poor is denoted as 15 to 26 points [15].

Credibility was assessed using the JAMA benchmark criteria. The JAMA criteria is a set of four categories, including authorship, attribution, disclosure, and currency, worth one point each [16].

Lastly, the Flesch Reading Ease (FRE) test and Flesch-Kincaid Grade Level (FKGL) analyzed the text within the video or the caption to assess readability. FRE uses four elements consisting of average sentence length in words, average word length in syllables, the average percent of “personal words”, and the average percent of “personal senses” [17]. Higher scores indicate easy-to-read material. The reading scores were then used to calculate reading grade level [18].

All statistics were performed using Microsoft Excel (2016) and IBM SPSS Statistics for Windows, Version 28 (IBM Corp., Armonk, NY, USA). Pearson’s correlation coefficient was calculated to determine relationships between each of the aforementioned parameters.

## Results

The application, TikTok, was searched using multiple hashtags, including “#trach,” “#tracheostomy,” “#trachea,” and “#tracheotomy” for relevant videos uploaded to the platform until October 15, 2021. The first 60 videos that satisfied the inclusion criteria were selected for this study. The total views for all videos were 17,712,281. Total likes were 693,812. Videos were primarily posted by individuals who did not identify themselves as healthcare professionals (72%) while the remainder were posted by physicians (10%), nurses (15%), respiratory therapists (1.7%), and emergency medical services (1.7%). In terms of views, videos posted by physicians made up 63% of all views while the rest of the views came from posts created by non-healthcare professionals (30%), nurses (3%), emergency medical services (4%), and respiratory therapists (0.01%).

The average DISCERN score was 24.83 for each of the videos, which correlates to a very poor score indicating serious or extensive shortcomings. The intra-class correlation for the DISCERN score between raters was 0.95 (95% CI: 0.54-0.99) indicating excellent reliability. The average JAMA score was 1.25 meaning only 1 out of the following 4 categories: authorship, attribution, disclosure, and currency, were identified in the videos. The average Flesch Reading Ease score was 70.59, indicating that the text within the video or in the caption was fairly easy to read. The average Flesch-Kincaid Grade level was 5.5.

Pearson’s correlation coefficient was calculated between each of the parameters and is represented in Table 1. There was a statistically significant positive correlation between the DISCERN score and views with R = 0.255 (p = 0.049), a positive correlation between DISCERN and likes R = 0.334 (p = 0.009), a positive correlation between DISCERN and JAMA R = 0.56 (p<0.001), and positive correlation between DISCERN and Flesch-Kincaid Grade Level R = 0.330 (p=0.010) and a negative correlation between DISCERN and Flesch Reading Ease Score R = -0.337 (p=0.009). There was a statistically significant positive correlation between JAMA and Flesch-Kincaid Grade Level R = 0.260 (p=0.045). See Appendices for more information.

## Discussion

The purpose of this study was to perform a cross-sectional analysis of the quality, credibility, and readability of TikTok tracheostomy videos to assess the adequacy of the information for patient and parental educational purposes. The quality, credibility, and readability were determined using the DISCERN scoring system, the JAMA benchmark criteria, and the Flesch Reading Ease score and Flesh-Kincaid Grade Level.

The results of this study indicate that the majority of the TikTok videos had serious or extensive shortcomings (average DISCERN score of 24.83). These results align with several other studies that have also shown a lack of quality content on TikTok [5,7,19]. Although several studies have demonstrated the lack of quality, the content is being disseminated to millions of viewers, as there was a total of 17 million views in the 60 videos analyzed in this study. Interestingly, even though physicians made up a total of 10% of the videos analyzed, physician posts generated 63% of all the total views. This means that physicians and other healthcare professionals have the ability and viewership to provide accurate and quality information to the public. Studies have shown that content produced by physicians has a greater probability of being reliable than educational videos posted by other sources [20]. It is imperative that quality content is produced and disseminated on platforms such as TikTok as large amounts of erroneous content already exist [1]. This erroneous content could be misleading to patients and other healthcare professionals leading to negative effects such as unnecessary concern, confusion, and mistrust [21].

We found a positive correlation between DISCERN and views, likes, and JAMA benchmark criteria, which indicates that videos with higher quality have higher credibility and generate more views and likes. The negative correlation between DISCERN and Flesch Reading Ease Score, positive correlation between DISCERN and Flesch-Kincaid Grade Level, and a positive correlation between JAMA and Flesch-Kincaid Grade Level indicate that higher quality, more credible videos tend to be more difficult to read. These results are important as they indicate a potential benefit of making higher-quality videos with an appropriate reading level. We found that higher-quality videos generated more exposure in the form of views and likes. The potential downside to these higher quality videos is that we found that they are harder to read, which could make information more difficult to understand, and identify an area for improvement for healthcare professionals who wish to develop content for TikTok. 

This study provides insight into the relationship between TikTok videos and readability. Many of the videos on TikTok include captions or text as either a way to augment what is said in the video or as a way to provide further context to the video. In terms of readability, our results demonstrated that the Flesch Reading Ease score was 70.59 indicating that the text was fairly easy to read. The average Flesch-Kincaid Grade level was 5.5 supporting the Flesch Reading Ease score. These results should be taken into account when considering how to produce future health content on TikTok as complex medical terminology must be summarized and explained in a way that most users can understand.

The limitations of this study include the inability to generalize these results over time since TikTok is a platform that can quickly change and evolve. Our results were obtained from a cross-sectional analysis of content within a specific time period further limiting its generalizability. Additionally, it is difficult to generalize the results of this study to videos of high or excellent quality (DISCERN score >51), as the average video analyzed was of poor quality. Lastly, the subjective nature of the scoring systems could cause differences in multi-rater systems. However, inter-rater reliability for DISCERN was determined to be high in this study.

## Conclusions

The primary objective of this study was to evaluate the quality, credibility, and readability of TikTok videos related to the topic of tracheostomy. This is one of the first studies assessing educational content on TikTok within the field of otolaryngology. We found that the quality of the videos on TikTok regarding tracheostomy rated very poorly on the DISCERN quality index but included text that was fairly easy to read. Currently, medical videos on TikTok do not meet the quality metrics needed to properly educate the public and should not be used as a primary resource. However, TikTok’s growing popularity provides an opportunity for the medical community to disseminate higher-quality information and provide a positive influence on its viewers.

## Appendices

**Table 1:** Correlation analysis of TikTok video metrics and evaluation criteria

	DISCERN	JAMA	Flesch Reading Ease Test	Flesch-Kincaid Grade Level
Views	R = 0.255 (p = .049)	R= 0.222 (p = 0.088)	R= -0.14 (p = 0.285)	R = 0.094 (p = 0.474)
Likes	R = 0.334 (p = 0.009)	R= 0.057 (p = 0.663)	R= -0.077 (p = 0.557)	R = 0.050 (p = 0.703)
Flesch-Kincaid Grade Level	R = 0.330 (p = 0.010)	R= 0.260 (p = 0.045)	R= -0.94 (p = <0.0001)	
Flesch Reading Ease Test	R = -0.337 (p = 0.009)	R= -0.249 (p = 0.055)		
JAMA	R = 0.56 (p = <0.0001)			
